# Microvesicle-transferred mitochondria trigger cGAS-STING and reprogram metabolism of macrophages in sepsis

**DOI:** 10.1128/spectrum.00781-25

**Published:** 2025-09-04

**Authors:** Ting Ji, Ting-ting Zhao, Sheng-Ze Long, Cai-Zhou Wei, De-Yun Cheng, Juan Chen, Liang-Jian Kuang

**Affiliations:** 1 Department of Key Laboratory of Ningxia Stem Cell and Regenerative Medicine, Institute of Medical Sciences, General Hospital of Ningxia Medical University 74747, Yinchuan, Ningxia, China; 2 Department of Pulmonary and Critical Care Medicine, General Hospital of Ningxia Medical University 74747, Yinchuan, Ningxia, China; 3 Department of Pulmonary and Critical Care Medicine, West China Hospital of Sichuan University 34753, Chengdu, Sichuan, China; 4 Department of Pulmonary and Critical Care Medicine, Guangxi Hospital Division of The First Affiliated Hospital, Sun Yat-sen University 678504 , Nanning, Guangxi, China; End TB Dx Consulting LLC, San Diego, California, USA

**Keywords:** sepsis, mitochondria, metabolism, metformin, microvesicles

## Abstract

The inflammatory cytokine storm is a hallmark of sepsis and is highly correlated with organ injury. Therefore, inhibiting inflammatory cytokine production is a straightforward strategy for effectively treating this disease. In this study, we found that microvesicles from lipopolysaccharide (LPS)-primed macrophages could transfer mitochondria to other macrophages and alter their biological functions. Microvesicles were isolated from LPS-primed macrophages and characterized by transmission electron microscopy. The function of microvesicle-transferred mitochondria in macrophages was evaluated by assessing the expression levels of inflammatory cytokines using immunofluorescent and quantitative real-time polymerase chain reaction (RT-qPCR) assays, and metabonomics using *in vitro* and *in vivo* models. Microvesicles derived from LPS-primed macrophages were able to transfer mitochondria to other macrophages. Functionally, these microvesicles induced classical activated macrophage (M1) polarization, reduced phagocytic capacity, altered mitochondrial homeostasis and metabolism in macrophages, and ultimately caused organ injury *in vivo*. Mechanistically, we demonstrated that metformin could inhibit the microvesicle-transferred mitochondrial reactive oxygen species (mtROS) and cyclic GMP-AMP synthase (cGAS)-stimulator of interferon genes (STING)-Interferon Beta (IFN-β) signaling activity, subsequently reducing inflammatory cytokine production. Our findings suggest that mtROS production is a critical cellular response in the inflammatory cytokine storm of sepsis, and the cGAS-STING-IFN-β signaling pathway may be a novel therapeutic target for sepsis treatment.

**IMPORTANCE:**

Sepsis remains lethal due to an uncontrolled “cytokine storm” damaging organs, yet specific treatments are lacking. Our study reveals a critical new mechanism: mitochondria transferred via microvesicles from stressed macrophages trigger this storm. These are transferred via microvesicles from stressed macrophages and trigger this storm. These transferred mitochondria reprogram recipient cells into damaging inflammatory (M1) states, reduce infection-fighting ability, disrupt metabolism, and cause organ injury. Importantly, we identify the mtROS/cGAS-STING-IFN-β pathway as the specific driver of inflammation within this process. Demonstrating that metformin blocks this pathway and reduces cytokine production reveals a novel strategy targeting the fundamental cause. This work is significant as it identifies mtROS/cGAS-STING-IFN-β as a key therapeutic target and repurposes metformin for potential sepsis treatment.

## INTRODUCTION

Sepsis remains the leading cause of death in intensive care units (1), driven by dysregulated immune responses and organ dysfunction (2). While current therapies fail to address the root pathophysiology, emerging evidence highlights the dual roles of cellular communication mechanisms in sepsis: they may exacerbate inflammation (pathological role) or serve as a therapeutic target (therapeutic potential).

This dualism underscores the need to systematically investigate pathways like mitochondrial transfer, which could bridge pathological mechanisms and clinical translation.

Platelets, neutrophils, and macrophages are central to sepsis pathogenesis. Platelets modulate adaptive immunity via MHC-I-CD8+T-cell interactions (3), while STING signaling in platelets drives septic thrombosis through granule secretion (4). Similarly, in addition, gasdermin D (GSDMD) in neutrophils mediates neutrophil extracellular traps (NETs) release, a process inhibited by disulfiram to reduce organ injury (5). Macrophages further contribute via pyroptosis-induced coagulation, as monocyte depletion attenuates lethality in sepsis models (6). However, whether mitochondrial transfer via microvesicles (MVs) represents a pathogenic amplifier or a therapeutic conduit remains unexplored.

MVs transfer mitochondria, RNA, and proteins between cells, enabling intercellular crosstalk (7). Macrophage-derived MVs have been shown to transfer miR-223 for macrophage differentiation (8), while monocyte-derived MVs deliver mitochondria to endothelial cells, inducing TNF responses (9). Crucially, LPS-primed macrophage MVs may transfer mitochondria to naive macrophages, altering their functional phenotype—a mechanism implicated in sepsis-induced immunopathology. Here, we hypothesize that MV-mediated mitochondrial transfer activates the cGAS-STING-INF-β axis, driving M1 polarization and functional exhaustion, while metformin mitigates this via mtROS suppression.

The cGAS-STING axis bridges innate sensing and inflammation: cGAS detects mitochondrial DAMPs (10), and STING hyperactivation correlates with sepsis severity (11, 12). Zhang et al. linked STING to GSDMD cleavage and coagulation via ITPR1 inhibition (13), yet whether MVs directly activate this axis in macrophages during sepsis remains unknown. We posit that MVs act as pathogenic vectors, delivering mitochondria to amplify STING signaling, thereby promoting sepsis progression.

Metformin, an AMPK activator, improves mitochondrial function (14) and mitigates sepsis-induced inflammation (15–17). While metformin’s effects on cGAS-STING are uncharacterized, its mtROs-suppressing activity (16) suggests potential to inhibit MV-driven STing activation. This dual action—targeting both mitochondrial transfer (pathological) and STING hyperactivation (therapeutic)—positions metformin as a candidate for reprogramming sepsis-associated immunopathology.

In this study, we demonstrate that LPS-primed macrophage MVs transfer mitochondria to naive macrophages, activating cGAS-STING-IFN-β signaling and inducing M1 polarization. Mechanistically, mitochondrial reactive oxygen species (mtROS) from transferred mitochondria drive STING activation, exacerbating organ injury in murine sepsis. Notably, metformin abrogates this axis by reducing mtROS, restoring phagocytic capacity, and improving survival. Our findings redefine MV-mediated mitochondrial transfer as a pathogenic axis in sepsis while identifying metformin as a dual-modality therapy: it suppresses pathological STING activation while preserving mitochondrial quality for tissue repair.

## MATERIALS AND METHODS

### Mice

Male C57BL/6J mice, aged 6–8 weeks (Co., Ltd., Shanghai, China), were housed in a specific pathogen-free environment at the Sichuan Huaxi Hospital Animal Laboratory (Chengdu, China). All animal experiments were conducted under guidelines approved by the Ethics Committee of Huaxi Hospital, affiliated with Sichuan University (Approval No: 20220422006).

### Cell culture

Raw 264.7 cells were obtained from the American Type Culture Collection and cultured in Dulbecco’s modified Eagle medium (DMEM) with 10% fetal bovine serum (FBS), supplemented with 2 mM L-glutamine, 1 mM sodium pyruvate, 100 U/mL penicillin, and 100 U/mL streptomycin. Bone marrow-derived macrophages (BMDMs) were cultured as previously described (18). Briefly, bone marrow from the femurs of 6- to 8-week-old mice was isolated and incubated with RPMI 1640 containing 10% fetal bovine serum, 2% antibiotic-antimycotic, and 10% L929 conditioned media. Medium was exchanged every 3 days, and on day 7, BMDMs were used for further experiments.

### Isolation of microvesicles from Raw 264.7 cells

Microvesicles were isolated from Raw 264.7 cells using a method described in a previous study (19). Briefly, Raw 264.7 cells were treated with LPS (100 ng/mL) or PBS for 24 h before the supernatant was harvested. The sample was centrifuged (260 × *g*, 5 min, 4°C) to remove cell debris, and the supernatant was further centrifuged at 20,000 × *g* for 30 min (4°C). The pellet containing MVs was resuspended in PBS and stored at −80°C for further experimentation. The dose of MVs used in experiments was based on the cell number of Raw 264.7 cells. For *in vitro* experiments, MVs were isolated from 1.2 × 10^8^ cells. For *in vivo* experiments, MVs were isolated from 2.4 × 10^8^ cells.

### Isolation of mitochondria from microvesicles

Mitochondria were isolated from BMDM cells using the Cell Mitochondria Isolation Kit (Beyotime, Jiangsu, China) according to the manufacturer’s instructions. Briefly, cells were collected, washed with PBS, and suspended in ice-cold isolation buffer for 15 minutes. After homogenization, the homogenate was centrifuged at 600 × *g* for 10 minutes at 4°C, and the supernatant was centrifuged at 11,000 × *g* for 10 minutes at 4°C. The mitochondria were collected in the pellet.

### Transmission electron microscopy

Microvesicle samples were diluted five times with PBS and applied to 200-mesh nickel grids. Samples were stained with 2% phosphotungstic acid for 5 minutes at room temperature and air-dried. Microvesicles were observed using a transmission electron microscope (TEM) (HT HT7800, Japan) at 80 kV.

### ZetaView nanoparticle tracking analysis

Purified microvesicles were diluted with PBS to measure particle size and concentration. The corresponding software, ZetaView (Germany), was used for data analysis. Dilution factors and resuspension volumes were used to convert the yield from concentration to an accurate number of particles.

### Mitochondrial transfer assay

Mitochondria from LPS-primed macrophages (Raw 264.7 cells) were transferred to BMDMs by MVs, as detected by mitochondria-associated probes according to a previous study (20). A MitoTracker Deep Red FM fluorescent probe was used to label active mitochondria in MVs derived from LPS-primed Raw 264.7 cells (LPS-MVs). Raw 264.7 cells were washed with PBS to remove residual FBS before staining, and 200 nM of MitoTracker Deep Red FM was added to incubate in the dark for 45 minutes. Cells were washed five times with PBS and treated with LPS (100 ng/mL) for 24 h before being collected for MV isolation. BMDMs at a density of 1 × 10^5^ were plated on a confocal dish under regular cell culture conditions to allow cells to adhere to the dish. BMDMs were washed with PBS and stained for endogenous mitochondria by incubation with 200 nM of MitoTracker Green for 45 minutes in the dark. After BMDMs were washed five times with PBS, MVs were added, and cells were cultured for an additional 24 h. The co-localization of endogenous and externally transferred mitochondria was observed by confocal microscopy.

### BMDMs engulf microvesicles

MVs were double-stained for confirmation. Briefly, the following steps were involved. (i) Extract MVs from RAW 264.7 and stain them with PKH-67. Prepare BMDM cell slides in advance and treat with PKH-67-stained MVs for 24 hours. (ii) Wash cells twice with 1× PBS (pH 7.4) preheated at 37°C. Fix cells in 4% formaldehyde solution at room temperature for 10–30 minutes. (iii) Wash cells 2–3 times and permeabilize them with 0.5% Triton X-100 solution for 5 minutes. (iv) Wash cells 2–3 times, apply TRITC-labeled phalloidin cyclic peptide working solution, cover the cells on the cover glass, and incubate at room temperature in the dark for 30 minutes. (v) Wash the cover glass with PBS three times, each for 5 minutes. Use DAPI solution to counterstain the cell nucleus, wash the cover glass with PBS, and invert it onto a glass slide with one drop of Fluoromount-G water-soluble sealing agent. (vi) Perform fluorescence observation under a confocal microscope.

### Measurement of macrophage dysfunction

For abnormal phenotype markers, the expression of M1 markers (e.g., IL-1β, IL-6, and TNF-α) and the expression of M2 markers (e.g., Arg1 and Retnal) were detected by qRT-PCR. The phagocytic deficiency was detected by immunofluorescence and Wb (phagocytic deficiency: measure by >50% decrease vs control). In addition, the mitochondrial ROS overproduction was detected by immunofluorescence.

### Measurement of mitochondrial membrane potential

Mitochondrial membrane potential analysis was performed using 5,5′,6,6′-tetrachloro-1,1′,3,3′-tetraethylbenzimidazolylcarbocyanine iodide (JC-1) dye according to a previous study (20). Briefly, BMDMs (1 × 10^4^) were adhered to a 24-well plate and stimulated with PBS, PBS-MVs, or LPS-MVs for 24 h. After treatment, cells were washed with PBS three times, and JC-1 (C2006, Beyotime, China) (20 µM) was added to the cells. After staining, wells were washed three times with 1 × dilution buffer. The intensity of red and green fluorescence of each image was measured using a confocal fluorescence microscope (Leica, Germany).

### Detection of mitochondrial superoxide production

After BMDMs (1 × 10^5^) were adhered to a 24-well plate, cells were stimulated with PBS, PBS-MVs, or LPS-MVs. After 24 h of stimulation, cells were washed with PBS three times, and MitoSOX (Thermo Fisher M36008) (5 µM) was added to the medium at 37°C for 20 minutes. Cells were washed with PBS three times, and mitochondrial superoxide production was assessed by flow cytometry.

### Detection of mitochondrial oxygen consumption rate

The oxygen consumption rate (OCR) assay was conducted using a BBoxiProbe R01 kit (Bestbio, China) to evaluate cell metabolism and mitochondrial state. Briefly, the following steps were involved. (i) Cells were cultured in a 96-well black plate with a transparent bottom and treated with LPS-MVs and PBS-MVs for 24 hours. (ii) The growth medium was refreshed, and 5 µL of oxygen fluorescent probe was added. (iii) Oxygen-blocking buffer was added to prevent external oxygen. (iv) Subsequently, the cell plate was used to detect changes in extracellular O2 consumption by fluorescence. The excitation wavelength was 455–468 nm, and the emission wavelength was 603 nm. Fluorescence intensity was measured every 3 minutes for 1.5 hours.

### *In vitro* phagocytosis assay of apoptotic cells and NETs

Bone marrow neutrophils were isolated from C57/BL6 mice, and neutrophil apoptosis and NET formation were induced according to previous studies (21, 22). For the phagocytosis assay, apoptotic neutrophils (20 µL/1 × 10^5^ in 100 µL) and NETs (20 µL/1 × 10^5^ in 100 µL) were added to BMDMs (1 × 10^4^) in 6-well plates at 37°C for 24 h. Immunofluorescence was used to assess the phagocytosis of BMDMs.

### Western blot analysis

Raw 264.7 cells (1 × 10^7^) and BMDMs (1 × 10^6^) were co-cultured with LPS-MVs, PBS-MVs, and PBS for 48 h before lysates were prepared for Western blotting. The BCA assay was used to determine protein concentration. Proteins were subjected to 10% SDS-PAGE before transfer to PVDF membranes. Membranes were blocked with 5% BSA and incubated with primary antibodies overnight at 4°C: anti-cGAS (1:1,000, ab252416, Abcam), anti-STING (1:1,000, ab288157, Abcam), anti-SIRPA (1:1,000, SAB33486), and anti-β-actin (1:1,000, T40104, Abmart). Anti-rabbit secondary antibodies (1:50,000, Ha1031, HUABIO) were used for detection on the ChemiDoc MP Imaging System (BIORAD, USA).

### Quantitative real-time polymerase chain reaction

Total RNA isolation from Raw 264.7 cells (1 × 10^7^) and BMDMs (1 × 10^6^) was performed using the Total RNA Extraction Kit according to the manufacturer’s instructions (Is1040, Promega USA). PCR was performed using SYBR green master mix (Yeason, China) on the StepOnePlus Real-Time PCR System (LightCycle480, ROCHE). The expression levels of target genes were normalized to GAPDH (1:1,000, T40104, Abmart). Primer sequences were as follows in Table S1.

### Histopathological and immunohistochemical analysis

After euthanizing the mice, sections from the lungs and livers were fixed in 4% paraformaldehyde (PFA), embedded in paraffin, sectioned, and stained with hematoxylin and eosin (H&E). Immunohistochemical staining for F4/80 (GB11027, Servicebio) was performed following a previously described protocol (23).

### Immunofluorescence

Immunofluorescence microscopy was used to detect TUNEL in the lungs and liver after mice were injected with PBS, PBS-MVs, and LPS-MVs. The sections of lungs and livers were incubated with a TUNEL Kit according to the manufacturer’s instructions (G1501, Servicebio). All samples were stained with DAPI for nuclear staining and assessed using immunofluorescence microscopy (Olympus, Japan).

### Cytokine production

Cytokines released by BMDMs and Raw 264.7 cells in the supernatant following different treatments (PBS, PBS-MVs, and LPS-MVs) were measured using IL-1β, IL-6, and TNF-α ELISA kits (NeoBioscience, China). Data were obtained using the Infinite M200 reader (Switzerland).

### Blood biomarkers of organ injury analysis

The animals were euthanized 24 hours after MV injection, and plasma samples were collected to measure creatine kinase-MB isoenzyme (CK-MB), alanine transaminase (ALT), aspartate transaminase (AST), and blood urea nitrogen (BUN), as described in a previous study (5).

### *In vivo* MV administration to WT C57BL/6 Mice

Four wild-type C57BL/6 mice in each group were injected with either PBS-MVs or LPS-MVs via the tail veins to evaluate the effect of MVs *in vivo* (for *in vivo* experiments, MVs were isolated from 2.4 × 10^8^ cells). Blood and tissues were collected 24 hours post-injection for further analysis.

### Treatment with metformin

Raw 264.7 cells and BMDMs were treated with PBS, PBS-MVs, and LPS-MVs in the presence or absence of metformin (1 mM) for 48 hours.

### Metabolic measurements

BMDMs were treated with either PBS-MVs or LPS-MVs for 48 hours. Afterward, the cells were detached and centrifuged at 800 *× g* for 5 minutes. The supernatant was discarded, and the cell pellets were stored at −80°C before being transported to Metabolon, Inc. (Durham, NC) for global metabolomic analysis. Abundance data for all metabolites were normalized to sample protein content, as measured by the Bradford assay, and the data are represented as relative normalized abundance. The data have been deposited in the OMIX database, China National Center for Bioinformation/Beijing Institute of Genomics, Chinese Academy of Sciences (https://ngdc.cncb.ac.cn/omix: accession no. OMIX001234).

### Statistical analysis

Data normality was assessed using Shapiro-Wilk tests. Normally distributed data are presented as mean ± SEM; non-parametric data as median (IQR). Multiple group comparisons were made with one-way ANOVA (parametric) or Kruskal-Wallis test (non-parametric), followed by *post hoc* testing with (Tukey/Dunnett/Sidak) correction for multiple comparisons. Significance levels are indicated as follows: *P < 0.05, **P < 0.01, ***P < 0.001, ****P < 0.0001. Adjusted *P*-values from *post hoc* tests are reported where applicable.

## RESULTS

### Release of mitochondrial content MVs and transfer to macrophages by LPS-stimulated Raw 264.7 cells

**Fig 1:**
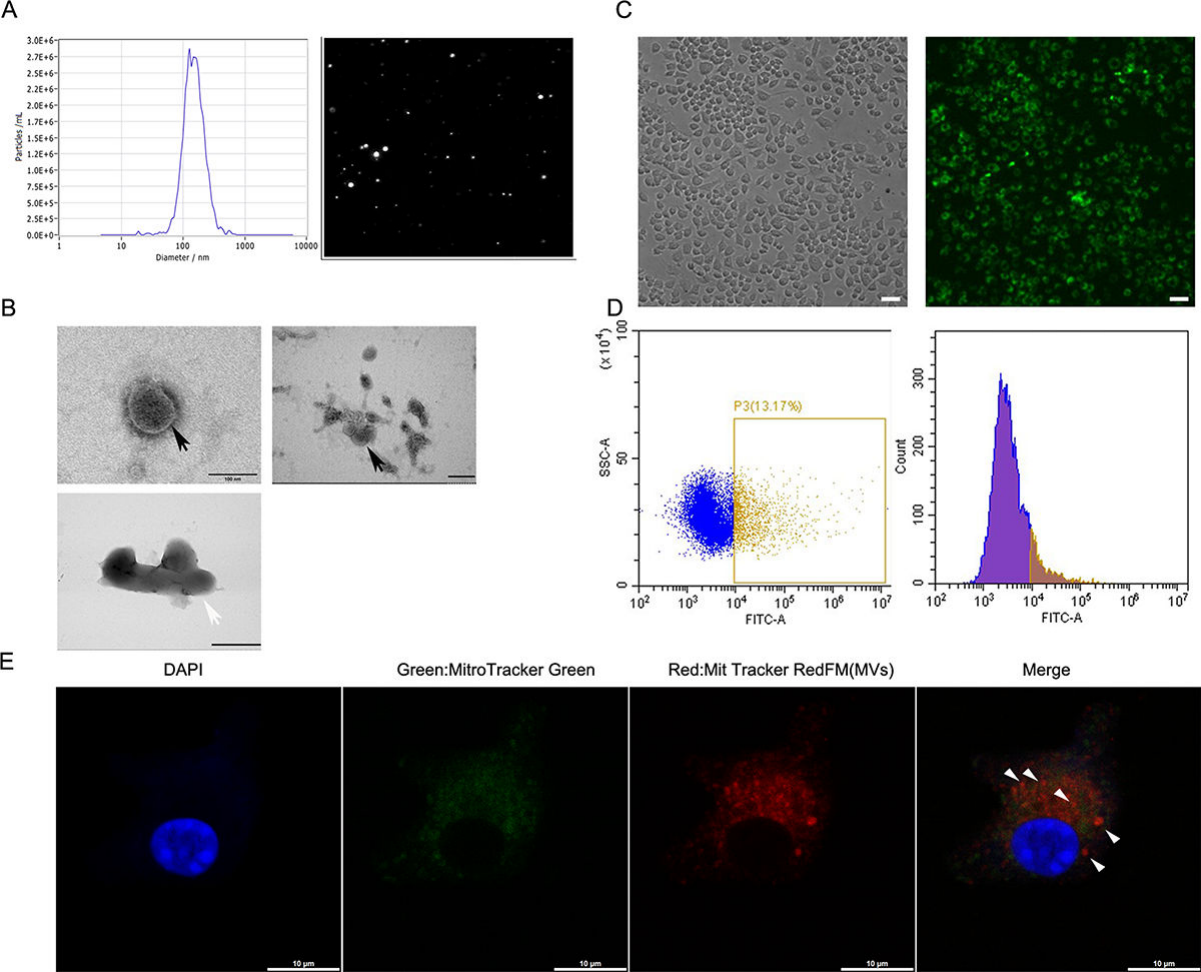
LPS-primed Raw 264.7 cells release microvesicles rich in mitochondrial content and can transfer to macrophages. (**A**) Shown are the size distributions of microvesicles isolated from PBS-MVs or LPS-MVs by nanoparticle tracking analysis (NTA). (**B**) Representative electron microscopy images of MVs. The pictures show microvesicles (black arrow) (scale bars, 100 nm [left] and 200 nm [right]) and mitochondria (white arrow) (scale bar, 500 nm). (**C and D**) Representative live images of LPS-activated MV (PKH26 green) internalization in macrophages (scale bar, 50 µm). (**E**) Representative live images of LPS-MV mitochondria internalization in BMDMs (scale bar, 10 µm).

Since mitochondrial proteins are known to be present in the MVs released by LPS-stimulated monocytes (24), we investigated whether these MVs could transfer mitochondria to other cells and affect the functions of recipient cells. Nanoparticle tracking analysis (NTA) demonstrated a particle size range of 100–1,000 nm for MVs (Fig. 1A). In addition, electron microscopy consistently showed the presence of MVs and vesicles containing mitochondria (Fig. 1B). Previous studies have shown that extracellular vesicles secreted from cells can be internalized by recipient cells. In this study, using immunofluorescence and flow cytometry, we observed significant uptake of MVs by Raw 264.7 cells after 24 hours of co-culture (Fig. 1C and D). Furthermore, we used PKH67 (Green) to stain microvesicles and phalloidin (Red) to label the cytoskeleton. Interestingly, we found that after BMDMs were cultured with microvesicles, green microvesicles were distributed in the cytoplasm but absent from the nucleus (Fig. S1).

Since mitochondria can be transferred between cells, we evaluated whether MVs could mediate mitochondrial transfer between macrophages. LPS-MVs were pre-treated with Mitotracker Deep Red before being co-cultured with BMDMs pre-stained with Mitotracker Green to visualize endogenous mitochondria. As expected, mitochondria from LPS-MVs were taken up by BMDMs (Fig. 1E).

### Mitochondria released by LPS-MVs induce mitochondrial dysfunction in macrophages

**Fig 2:**
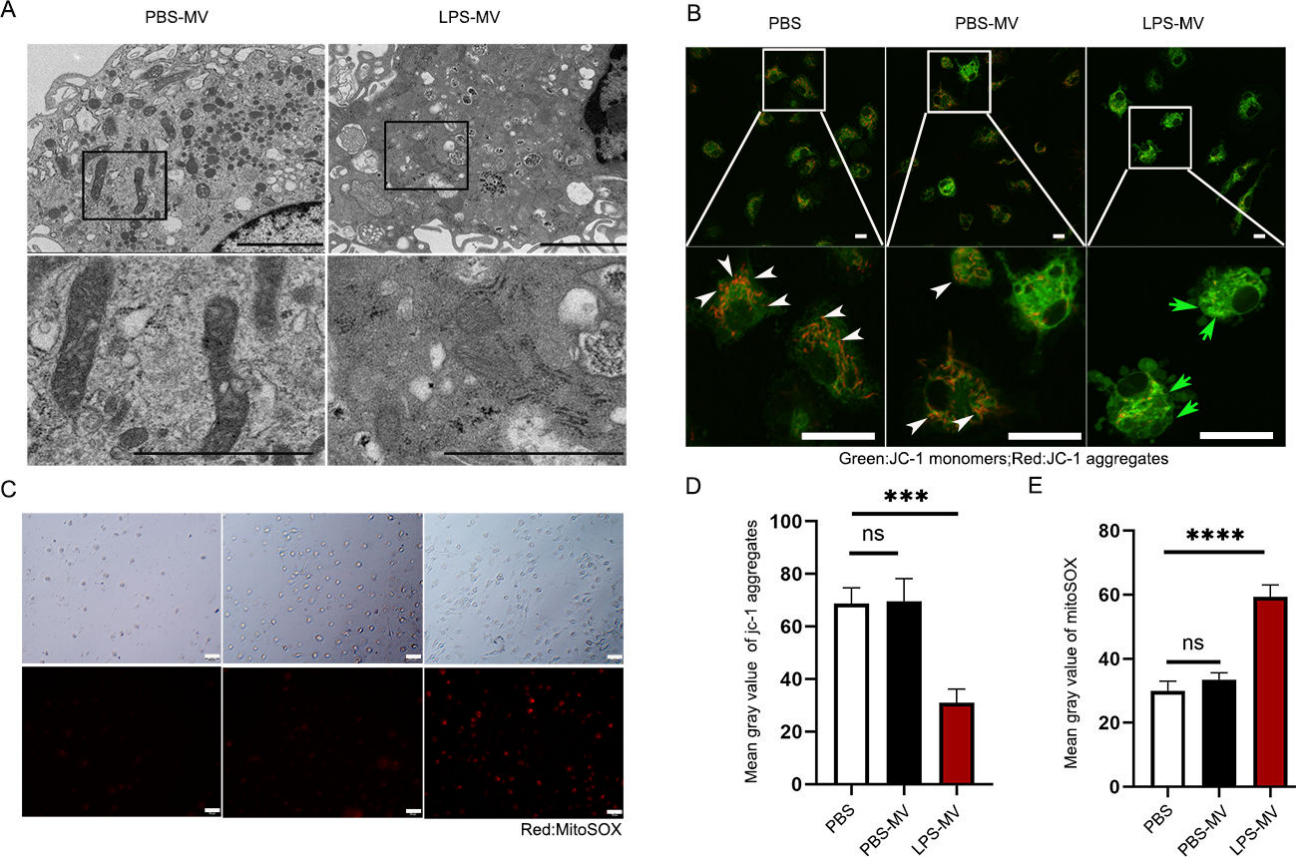
Lipopolysaccharide (LPS)-primed macrophage releasing MVs induces mitochondrial dysfunction in macrophages. (**A**) Mitochondria in BMDMs from PBS-MV and LPS-MV treatment assessed by TEM (scale bar, 2 µm). (**B**) Representative confocal microscopy of JC-1 dye fluorescence as an indicator of mitochondrial membrane potential in BMDMs after exposure to PBS, PBS-MVs, and LPS-MVs for 24 h. White arrows indicate the areas of depolarized mitochondrial membranes (JC-1 monomers), and green arrows indicate polarized mitochondrial membranes (JC-1 aggregates) (scale bar, 20 µm). (**C**) Representative live images of BMDMs in mitochondrial superoxide production detected with MitoSOX after exposure to LPS-primed MVs and PBS-primed MVs for 24 h (scale bar, 50 µm). (**D**) Quantification of the mean gray value of JC-1 expression on BMDM after treatment with PBS, PBS-MV, and LPS-MV. (**E**) Quantification of the mean gray value of MitoSOX after treatment with PBS, PBS-MVs, and LPS-MVs for 24 h.

Given that mitochondria regulate cell function, we investigated whether LPS-MVs could alter mitochondrial function in macrophages. Using TEM, we found that the mitochondrial structure was clear in the presence of PBS-MVs but obscure in the presence of LPS-MVs. In addition, the number of mitochondria in macrophages exposed to LPS-MVs was lower than in those exposed to PBS-MVs (Fig. 2A). Using immunofluorescence, we examined the mitochondrial membrane potential of macrophages in the presence of LPS-MVs or PBS-MVs. A significantly lower mitochondrial membrane potential was observed in cells cultured with LPS-MVs compared to those with PBS-MVs or PBS (Fig. 2B and D). No difference was detected between PBS-MVs and PBS treatments. Furthermore, we isolated mitochondria from LPS-MVs and co-cultured them with BMDMs. These mitochondria induced a lower mitochondrial membrane potential compared to those from PBS-MVs or PBS (Fig. S2B). We also examined the production of mtROS. As expected, mtROS production increased in macrophages cultured with LPS-MVs but not with PBS-MVs or PBS (Fig. 2C and E). No difference was identified between PBS-MVs and PBS treatments. We also measured the oxidative respiration capacity (ORC) as an indicator of mitochondrial function. LPS-MVs reduced the level of OCR in macrophages, while PBS-MVs and PBS did not (Fig. S3A and B). These results support the hypothesis that mitochondrial transfer alters mitochondrial function in recipient macrophages.

### LPS-MVs induce the M1-like macrophage phenotype and reduce phagocytic capacity in macrophages

**Fig 3:**
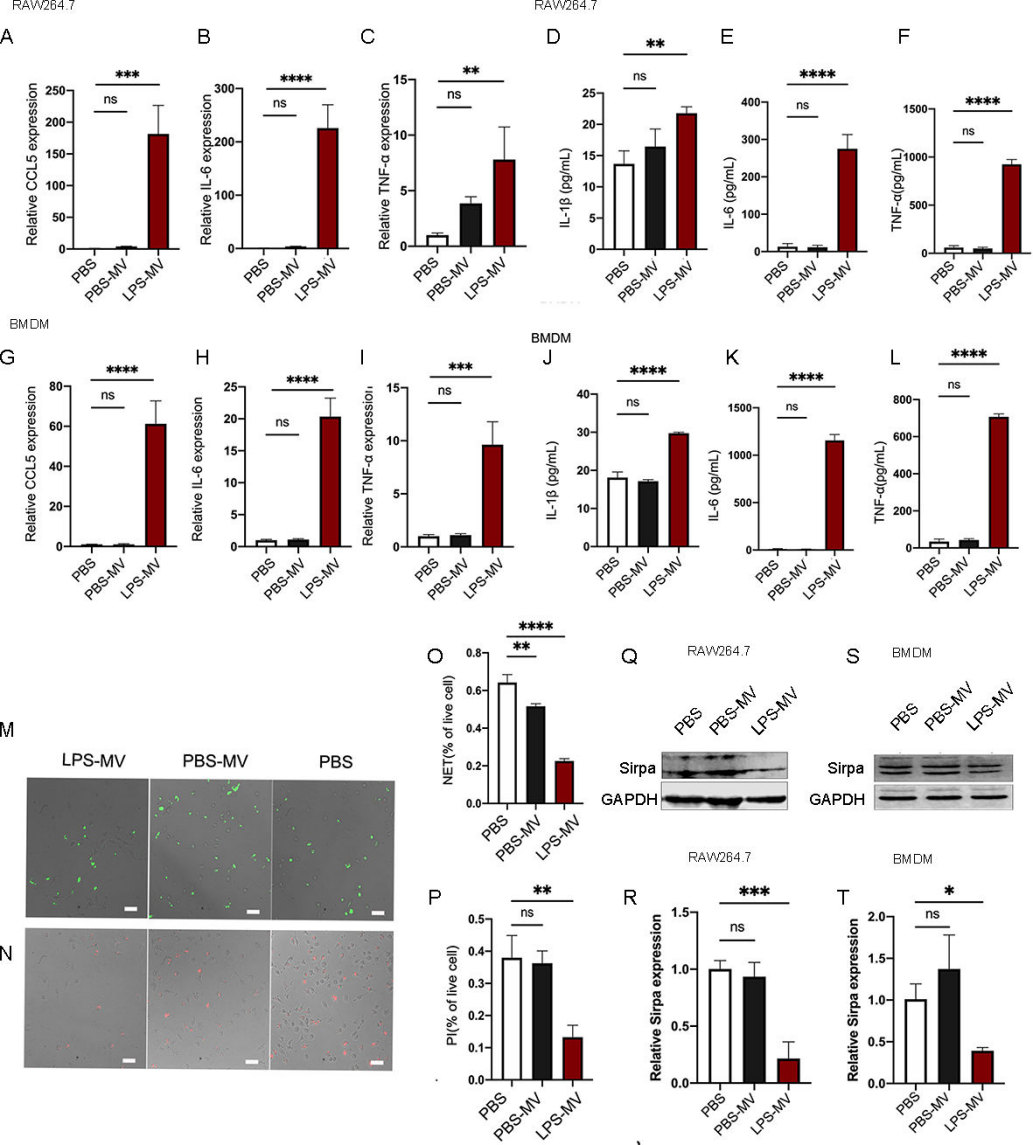
Microvesicles were purified from PBS-primed or LPS-primed macrophages and were then applied to recipient Raw 264.7 macrophages and BMDMs. Raw 264.7 cells were stimulated with PBS, PBS-MVs, and LPS-MVs for 24 h and harvested for mRNA detection. CCL5 (**A**), IL6 (**B**), and TNF-α (**C**) expressions were quantified by qRT-PCR. Correspondingly, supernatants of stimulated cells (**A–C**) were collected, and the releases of IL-1β (**D**), IL-6 (**E**), and TNF-α (**F**) were quantified (ELISA). Data are presented as means ± SEMs. BMDMs were stimulated with PBS, PBS-MVs, and LPS-MVs for 24 h and harvested for mRNA detection. CCL5 (**G**), IL6 (**H**), and TNF-α (**I**) expressions were quantified by qRT-PCR. Correspondingly, supernatants of stimulated cells (**G–I**) were collected, and the releases of IL-1β (**J**), IL-6 (**K**), and TNF-α (**L**) were quantified (ELISA). Data are presented as means ± SEMs. Ns, not significant; *P < 0.05; **P < 0.01; ***P < 0.001; ****P < 0.0001. Data are presented as means ± SEMs of three independent experiments. One-way ANOVA was used to analyze the *P* values of the different groups. For the detected phagocytic capacity of macrophages, BMDMs were cultured with PBS, PBS-MVs, and LPS-MVs for 48 h. This was followed by the addition of Sytox Green-labeled NET and PI staining of apoptotic neutrophils into BMDMs for 24 h. (**M**) Representative image and (**O**) quantification of phagocytic NETs in BMDMs. (**N**) Representative image and (**P**) quantification of phagocytic apoptotic neutrophils in BMDMs. (**Q**) The expression of Sirpa on Raw 264.7 cells after treatment with PBS, PBS-MVs, and LPS-MVs was assessed by western blotting. (**R**) Quantification of SIRPA expression on Raw 264.7 cells after treatment with PBS, PBS-MVs, and LPS-MVs for 48 h. (**S**) The expression of Sirpa on BMDM after treatment with PBS, PBS-MVs, and LPS-MVs was assessed by western blotting. (**T**) Quantification of SIRPA expression on BMDMs after treatment with PBS, PBS-MVs, and LPS-MVs for 48 h. Data are presented as means ± SEMs. Ns, not significant; *P < 0.05; **P < 0.01; ***P < 0.001; ****P < 0.0001. Data are presented as means ± SEMs of three independent experiments. One-way ANOVA was used to analyze the *P* values in the different groups (scale bar, 50 µm).

Next, we evaluated macrophage polarization markers to assess whether mitochondrial transfer from MVs regulated recipient macrophage polarization (25). Examination of macrophage polarization markers in Raw264.7 cells and BMDMs showed significantly increased expression of M1-like macrophage polarization marker genes (CCL5, IL-6, and TNF-α) (Fig. 3A through C and G through I), while the expression of M2-like macrophage polarization marker genes remained unchanged (Fig. S4A and B). These results indicate that LPS-MVs induced M1 polarization. Interestingly, we did not detect any differences in gene expression between control macrophages and PBS-MV-treated macrophages. These results suggest that LPS-MVs could induce macrophages toward an inflammatory M1 phenotype but not an M2 phenotype.

To functionally confirm the M1 macrophage phenotype transition, we evaluated the levels of inflammatory cytokines (IL-1β, IL-6, TNF-α), as macrophages are the major sources of inflammatory cytokines in sepsis. As expected, increased production of inflammatory cytokines was observed in LPS-MV-treated macrophages compared to untreated or PBS-MV-treated macrophages (Fig. 3D through F and J through L). These results imply that LPS-MVs induce M1 macrophage polarization and inflammatory cytokine production, which may contribute to the inflammatory cytokine storm during sepsis.

Since apoptotic neutrophils (26) and NETs (27) are present during sepsis, we also evaluated the ability of macrophages to engulf apoptotic neutrophils and NETs in the presence of PBS-MVs or LPS-MVs. Notably, the capacity of macrophages to engulf NETs was reduced after treatment with LPS-MVs compared to PBS-MVs or PBS (Fig. 3M and O). Similarly, the macrophage capacity to engulf apoptotic neutrophils was impaired in the presence of LPS-MVs (Fig. 3N and P). Signal regulatory protein α (SIRPA) has been shown to regulate macrophage phagocytic capacity (28). We assessed SIRPA expression in macrophages in the presence of PBS-MVs or LPS-MVs and found that SIRPA expression was downregulated in Raw 264.7 cells (Fig. 3Q and R) and BMDMs (Fig. 3S and T) compared to PBS and PBS-MVs. These results indicate that LPS-MVs impair the phagocytic capacity of macrophages, likely due to the downregulation of SIRPA. This could explain why apoptotic neutrophils and NETs accumulate during sepsis, contributing to organ injury and thrombogenesis.

### Metformin inhibits LPS-MV-triggered cGAS-STING-IFN-β signaling pathways in macrophages

**Fig 4:**
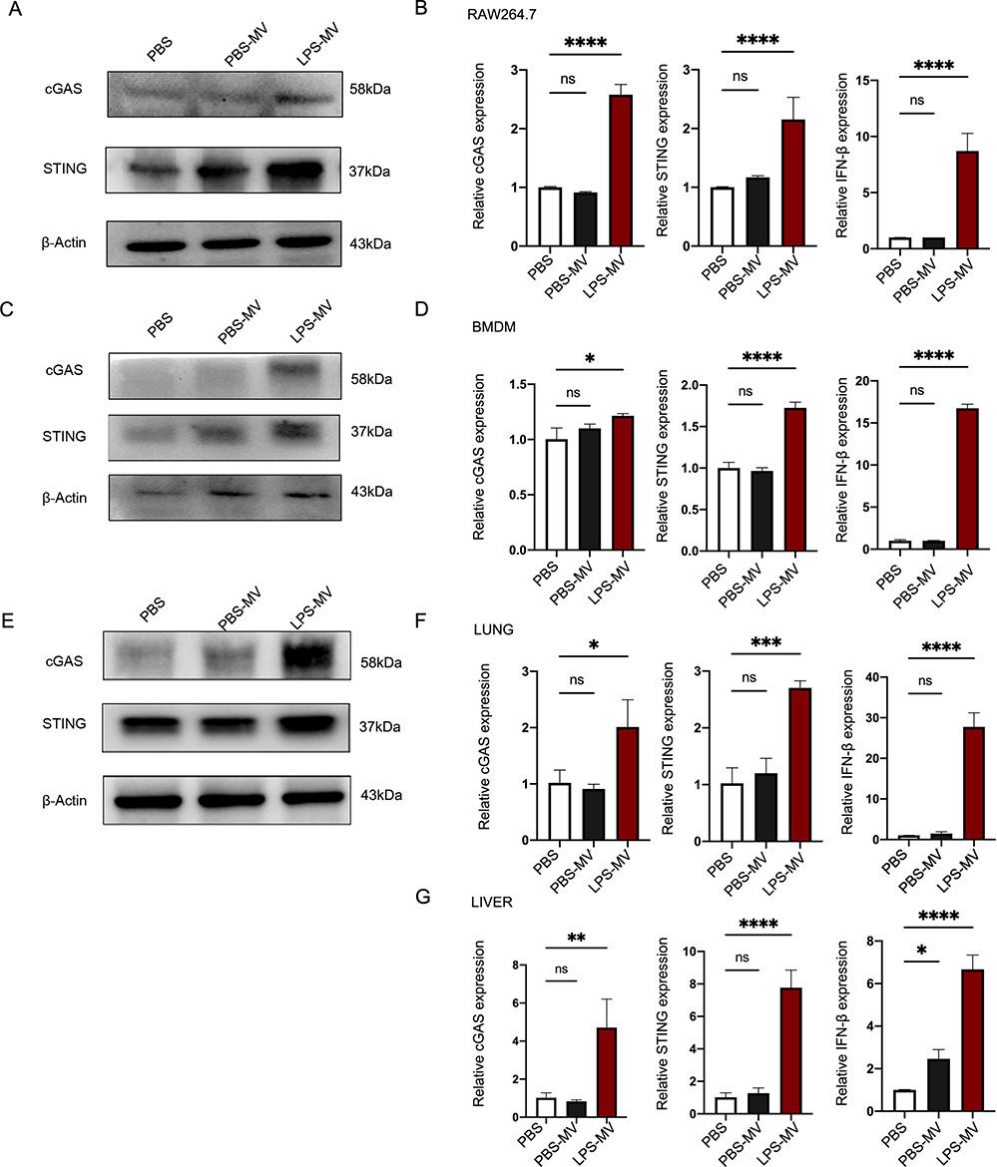
LPS-MVs trigger cGAS-STING-IFN-β signaling pathways in macrophages (**A and B**) Raw 264.7 cells were stimulated with PBS, PBS-MVs, and LPS-MVs for 48 h. Cells were harvested and analyzed for cGAS and STING by immunoblotting (**A**), and mRNA preparation and cGAS, STING, and IFN-β gene expressions were quantified by qRT-PCR (**B**). BMDMs were stimulated with PBS, PBS-MVs, and LPS-MVs for 48 h. Cells were harvested and analyzed for cGAS and STING by immunoblotting (**C**), and mRNA preparation and cGAS, STING, and IFN-β gene expressions were quantified by qRT-PCR (**D**). Mice were injected with PBS, PBS-MVs, and LPS-MVs for 24 h. Lungs were harvested and analyzed for cGAS and STING by immunoblotting (**E**), and mRNA preparation and cGAS, STING, and IFN-β gene expressions were quantified by qRT-PCR (**F**). Mice were injected with PBS, PBS-MVs, and LPS-MVs for 24 h. Livers were harvested, and mRNA preparation and cGAS, STING, and IFN-β gene expressions were quantified by qRT-PCR (**G**). Data are presented as means ± SEMs. Ns, not significant; *P < 0.05; **P < 0.01; ***P < 0.001; ****P < 0.0001. Data are presented as means ± SEMs of three independent experiments. One-way ANOVA was used to analyze the *P* values in the different groups.

Mitochondrial damage and the leakage of mitochondrial DNA into the cytosol can activate the STING signaling pathway, leading to pathological inflammation (29). Therefore, we investigated whether LPS-MVs could affect this process. We first examined the expression of cGAS, STING, and IFN-β mRNA and proteins in macrophages following treatment with LPS-MVs and PBS-MVs. We observed increased expression of these genes at both the mRNA and protein levels in macrophages incubated with LPS-MVs compared to PBS-MVs or PBS (Fig. 4A through D). In addition, we found that LPS-MVs upregulated the expression of phosphorylated STING (Fig. S4A and B). These findings indicate that mitochondrial transfer can trigger the cGAS-STING-IFN-β signaling pathway. Importantly, consistent with *in vitro* observations, the expression of cGAS, STING, and IFN-β mRNA and proteins was also elevated in the lungs and livers of mice administered LPS-MVs compared to those injected with PBS-MVs or PBS (Fig. 4E through G).

**Fig 5:**
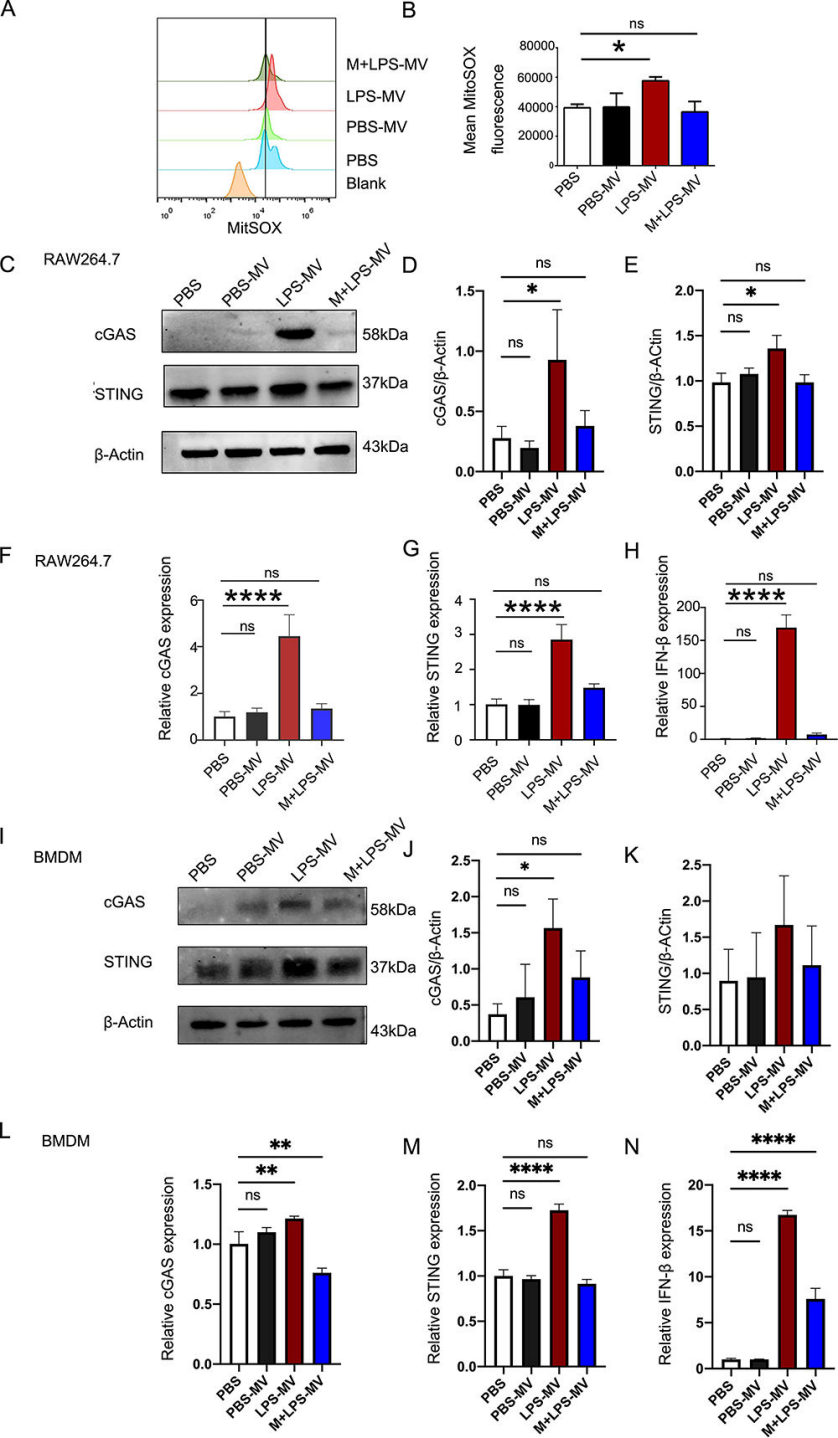
Metformin inhibits the pathway of cGAS-STING-IFNβ activation in macrophages by reducing mtROS production when exposed to LPS-MVs. (**A**) The production of mtROS in BMDMs was measured by flow cytometry after exposure to PBS-MVs, LPS-MVs, or PBS. (**B**) Quantification of mean fluorescence intensities of mtROS in different experimental conditions. (**C–E**) cGAS and STING in Raw 264.7 cells ± metformin were measured by WB when exposed to PBS-MVs, LPS-MVs, or PBS. (**F–H**) cGAS, STING, and IFN-β in Raw 264.7 cells ± metformin were measured by qPCR in different experimental conditions. (**I–K**) cGAS and STING in BMDMs ± metformin were measured by WB when exposed to PBS-MVs, LPS-MVs, or PBS. (**L–N**) cGAS, STING, and IFN-β in BMDMs ± metformin were measured by qPCR in different experimental conditions. Data are presented as means ± SEMs of three independent experiments. One-way ANOVA was used to analyze the *P* values in the different groups.

Since metformin has been reported to decrease mtROS production (30) and regulate the cGAS-STING signaling pathway, we investigated whether metformin could alter LPS-MV-triggered cGAS-STING signaling activation and mtROS production in macrophages exposed to LPS-MVs. We found that metformin significantly reduced mtROS production in macrophages exposed to LPS-MVs (Fig. 5A and B), accompanied by a reduction in cGAS, STING, and IFN-β mRNA and protein levels in both Raw 264.7 cells (Fig. 5C through H) and BMDMs (Fig. 5I through N). These data demonstrate that metformin inhibits the LPS-MV-activated cGAS-STING-IFN-β signaling pathway.

### Mitochondria released by LPS-MVs alter metabolism in macrophages

**Fig 6:**
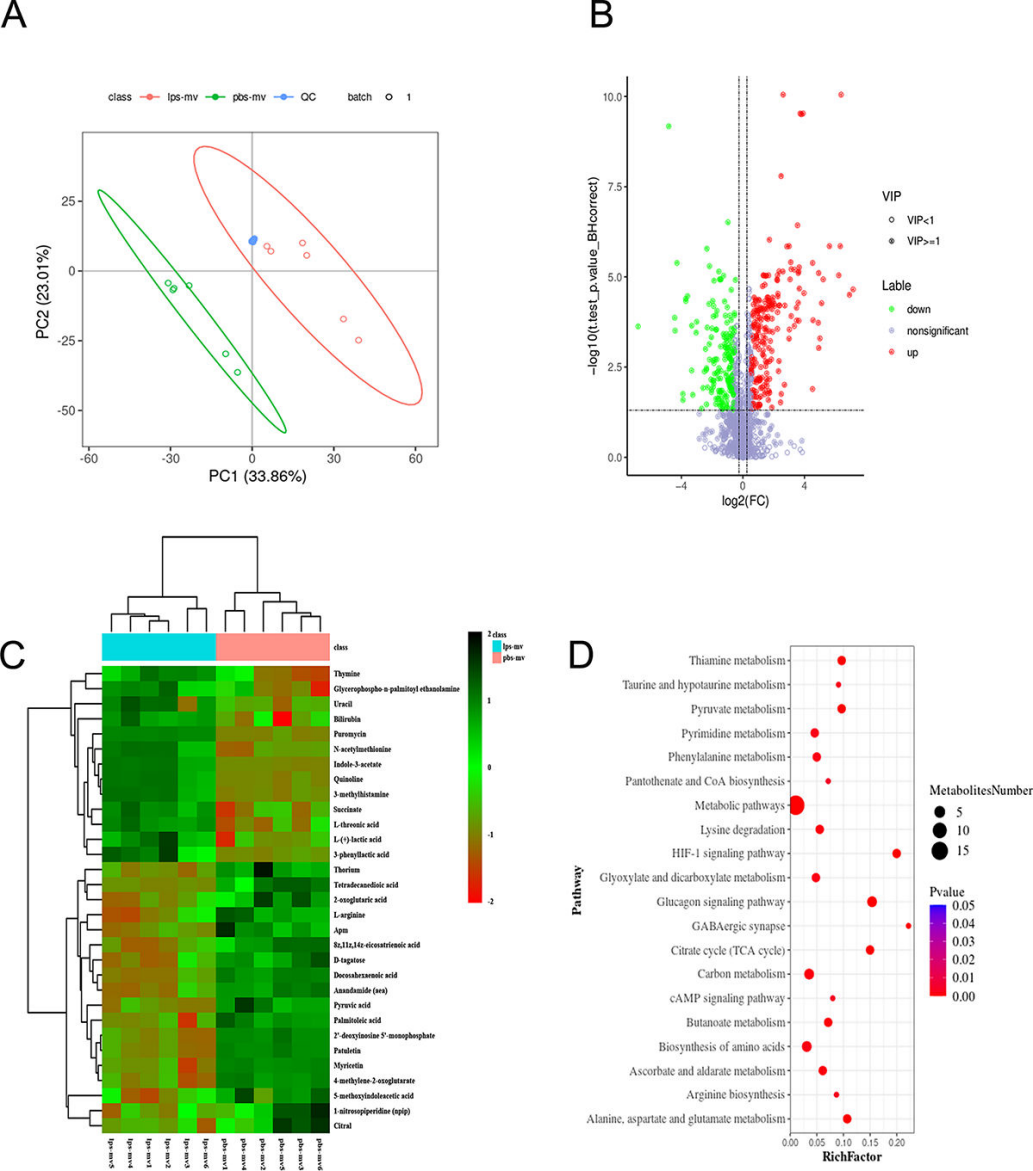
LPS-MVs regulate metabolism in macrophages (**A**) PCA showing clustering by treatment of metabolomes from BMDMs treated with PBS-MVs or LPS-MVs. (**B**) Volcano plot showing statistically significant metabolisms of BMDMs differentially expressed in the presence of PBS-MVs vs. LPS-MVs. (**C**) Heatmap of relative abundance of 31 metabolites in macrophages incubated in the presence of PBS-activated MV or LPS-activated MV. Each column represents an individual sample (*n* = 6/group). (**D**) Bubble plots of pathways with significant enrichments of differential metabolites.

Metabolism plays a critical role in sepsis (31). Recently, activated platelets have been shown to transfer mitochondria to mesenchymal stem cells and regulate metabolic reprogramming (32). To identify the metabolic pathways involved in macrophages due to mitochondrial transfer from LPS-MVs, we performed a non-targeted metabolic analysis of the culture supernatant following BMDM incubation with PBS-MVs or LPS-MVs. Principal component analysis (PCA) of total protein expression identified differential grouping of LPS-MV treatment compared to PBS-MV treatment, accounting for 33.86% of the variance in protein expression (Fig. 6A). Differential expression analysis identified 208 significantly upregulated and 177 significantly downregulated genes in BMDMs treated with LPS-MVs compared to PBS-MVs (Fig. 6B), at an FDR of <0.05 and a log2 fold-change of ≥1.2. These results demonstrate marked alterations in macrophages during LPS-MV treatment. A panel of 31 metabolites (mainly from the tricarboxylic acid [TCA] cycle, fatty acids, and glycolysis) was identified and used to construct a heatmap-based unsupervised hierarchical clustering analysis (Fig. 6C). The top 25 metabolites with the highest alterations are shown in Table S2. Integrative pathway analysis revealed significant downregulation of fatty acid metabolites, including tetradecanedioic acid, palmitoleic acid, docosahexaenoic acid, and 8z,11z,14z-eicosatrienoic acid, and upregulation of glycolysis (Fig. 6D). These results suggest that metabolism in macrophages was reprogrammed due to the transfer of mitochondria from LPS-MVs.

### LPS-MVs induce multiple organ injury and systemic inflammation in mice

**Fig 7:**
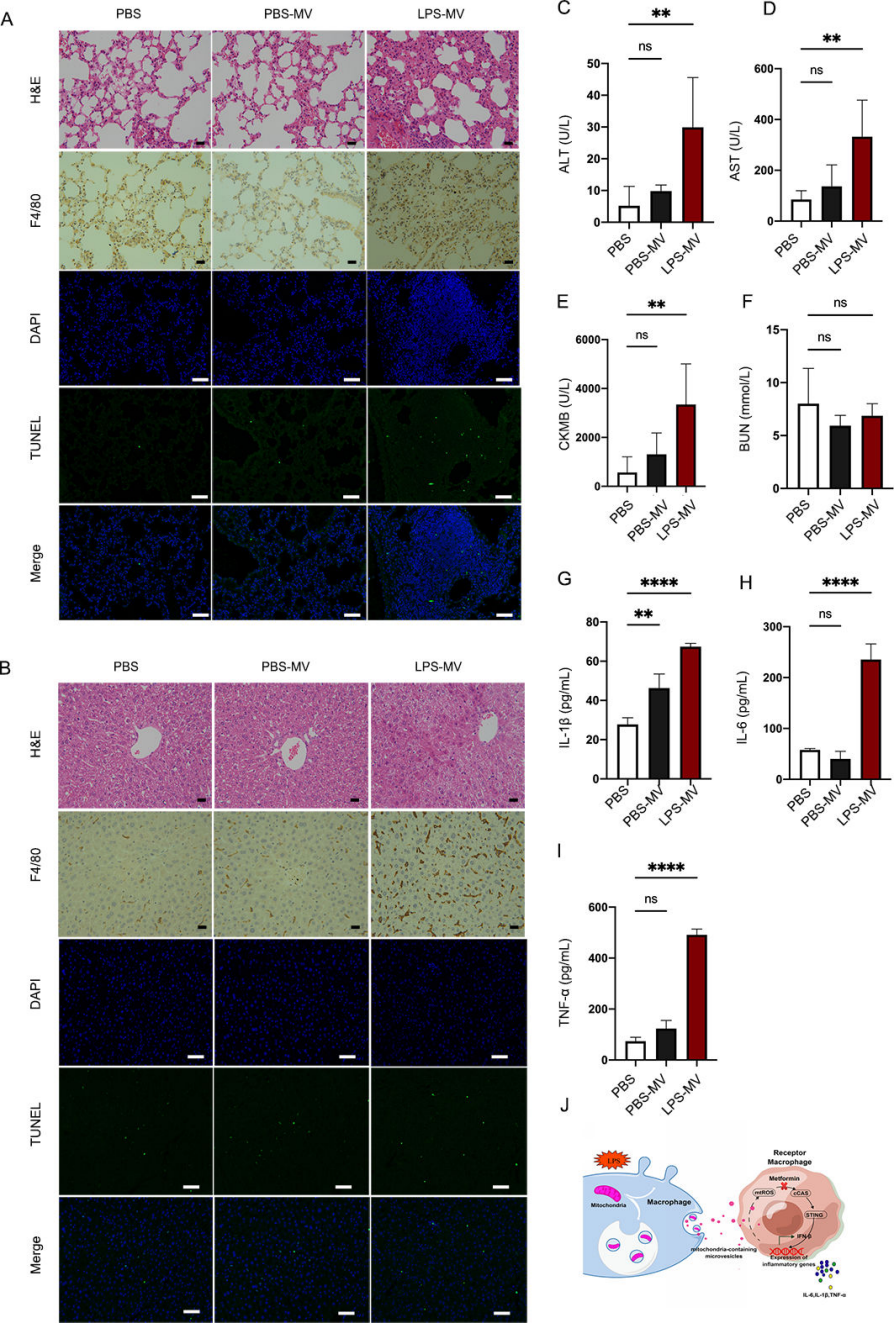
LPS-MVs induce organ injury and systemic inflammation *in vivo.* MVs were transferred to C57 mice through tail vein injection. Mice were euthanized 24 h after injection (*n* = 4/group). (**A and B**) Representative images of H&E staining of lung (**A**) and liver (**B**) sections from mice. F4/80 staining in the lungs and liver was detected by immunohistochemistry (scale bar, 20 µm). The expression of TUNAL in the lungs and liver was detected by immunofluorescence (DAPI, blue; TUNAL, green) (*n* = 3/group) (scale bar, 50 µm). (**C–F**) Circulating levels of organ injury markers: ALT (**C**); AST (**D**); CK-MB (**E**); and BUN (**F**) (*n* = 4/group). The systemic levels of IL-1β (**G**), IL-6 (**H**), and TNF-α (**I**) were detected by ELISA 24 h after mice were treated with PBS, PBS-MVs, and LPS-MVs (*n* = 4/group). Ns, not significant; *P < 0.05; **P < 0.01; ***P < 0.001; ****P < 0.0001. Data are presented as means ± SEMs of three independent experiments. One-way ANOVA was used to analyze the *P* values in the different groups. (**J**) Scheme of LPS-induced dysfunctional mitochondria in macrophages and microvesicles released during sepsis, resulting in the inflammation cytokine storm. During sepsis, macrophages exposed to LPS induce mitochondrial dysfunction. These dysfunctional mitochondria are translocated into lysosomes in the cytoplasm of macrophages and are released via microvesicles. Secreted microvesicles, which contain dysfunctional mitochondria, are further internalized and activate the mtROS/cGAS/STING pathways in recipient macrophages, resulting in the inflammation cytokine storm. However, metformin can inhibit the activation pathway.

To further evaluate the effect of LPS-primed MVs *in vivo*, we administered MVs to mice and examined organ injury and systemic inflammatory responses. Histopathological analysis of lung tissue revealed enhanced edema and thickening of alveolar septa (Fig. 7A). Considering that *in vitro* LPS-MVs could induce M1 macrophage polarization, we further evaluated macrophage infiltration into the lungs and liver in mice administered LPS-MVs compared to PBS-MVs or PBS. Indeed, F4/80+ macrophages infiltrating the lungs (Fig. 7A) and liver (Fig. 7B) were significantly increased in mice subjected to LPS-MV injection compared to those treated with PBS-MVs or PBS. No difference was identified between mice treated with PBS-MVs and PBS. In addition, a similar trend of cell apoptosis was observed as determined by TUNEL staining in the lungs (Fig. 7A) and liver (Fig. 7B). Biochemistry analysis also showed increased levels of biochemical markers of organ damage, specifically ALT (Fig. 7C), AST (Fig. 7D), and CK-MB (Fig. 7E), but not BUN (Fig. 7F) in mice receiving LPS-MVs compared to those injected with PBS-MVs or PBS. Consistent with these findings, significant increases in plasma concentrations of inflammatory cytokines IL-1β (Fig. 7G), IL-6 (Fig. 7H), and TNF-α (Fig. 7I) were also observed in mice receiving LPS-MVs. These results suggest that LPS-MVs can induce organ injury and increase the levels of inflammatory cytokines, implying that they induce macrophage infiltration and result in organ injury and inflammation *in vivo*. This study thus showed that LPS-induced macrophage-releasing mitochondria-containing MVs could trigger the mtROS/cGAS/STING/IFN-β pathway and induce inflammatory cytokine production. Notably, metformin can inhibit the activation of this signaling pathway (Fig. 7J).

## DISCUSSION

Sepsis is a common pathological condition characterized by an inflammatory cytokine storm, which contributes to multiple organ dysfunction (33). However, the mechanisms underlying the occurrence of inflammatory cytokine storms remain unclear. This study demonstrates a novel mechanism involving mitochondrial transfer and the activation of the mtROS-cGAS-STING-IFN-β pathway, along with metabolic reprogramming in macrophages. Our findings may support the development of novel treatments for inflammatory cytokine storms in sepsis.

Mitochondrial transfer by extracellular vesicles (EVs) can regulate mitochondrial function in recipient cells (34), and mitochondrial dysfunction contributes to the severity and outcome of septic shock (35). This study showed that after mitochondrial transfer from LPS-MVs, recipient macrophages exhibited fewer mitochondria, mitochondrial membrane potential damage, and increased mtROS production. Notably, overproduction of mtROS can disrupt mitochondrial membrane integrity and further damage the cell (36, 37).

Many studies have demonstrated that EV production increases in response to stimuli that induce cellular differentiation (38). Therefore, we investigated the functional impact of mitochondrial transfer on macrophages. We observed that mitochondrial transfer from LPS-MVs induced the expression of M1 macrophage markers, including CCL5, IL-6, and TNF-α. Notably, this study also showed that the adoptive transfer of MVs could induce macrophage polarization in the lungs and livers of mice. These findings align with those of Ismail et al. (8), who found that treating THP-1 cells with MVs from PMA-stimulated THP-1 cells significantly increased the surface expression of macrophage differentiation markers compared to vehicle-treated samples.

Furthermore, neutrophils and NETs play a critical role in the pathophysiology of sepsis. Some studies have demonstrated that the inflammatory alveolar environment of early acute respiratory distress syndrome (ARDS) initially delays apoptosis; however, these neutrophils ultimately undergo apoptosis in the alveoli. Notably, efferocytosis of apoptotic neutrophils by alveolar macrophages contributes to the resolution of inflammation (39). Recently, Mahida et al. (40) revealed that patients with sepsis and ARDS have significantly reduced alveolar macrophage efferocytosis compared to those without ARDS, which may contribute to the increased inflammation in sepsis-related ARDS. NETs not only kill bacteria but can also induce organ injury and thrombogenesis (41). In addition, Chiang et al. (42) reported that 13-series (T-series) resolvins (RvTs) were able to reduce NETosis and enhance macrophage NET clearance via the cyclic adenosine monophosphate-protein kinase (AMPK) axis, resulting in the resolution of excessive uncontrolled inflammation. This study showed that the phagocytic capacity of macrophages for apoptotic neutrophils and NETs was impaired in the presence of LPS-MVs. These phenomena are mainly attributed to mitochondrial dysfunction, as mitochondria are the powerhouses of immunity (43). In addition, SIRPA downregulation may have partly contributed to the results. However, our results differed from a previous study that reported a significant increase in phagocytic activity in macrophages from either GM-CSF or GM-CSF-derived MVs compared to freshly isolated monocytes (8). This variation may be caused by the different functions of MVs derived from cells primed with different stimuli, and whether LPS-MVs regulate AMPK activation requires further investigation.

Given that the release of inflammatory cytokines was increased in macrophages after LPS-MV treatment, we speculated that the uptake of dysfunctional mitochondria by active macrophages might modulate their inflammatory cytokine production. Supporting this hypothesis, we observed mitochondrial damage in macrophages in the presence of LPS-MVs, which might have released mitochondrial DNA into the cytosol. Subsequently, this DNA activated the cGAS-STING-IFN-β signaling pathway and induced excessive cytokine production, ultimately leading to an inflammatory cytokine storm. Interestingly, this pathway was inhibited after metformin treatment. Our findings were consistent with those of Chung et al. (44), who reported that after mitochondrial damage, mitochondrial DNA was released into the cytosol, which, in turn, activated the cytosolic cGAS-STING DNA-sensing pathway to induce immune cell recruitment and cytokine production. In addition, Saskia et al. observed that the release of membrane vesicles from the gut microbiota led to the systemic delivery of bacterial DNA to host cells. Subsequently, these MVs could trigger the cGAS-STING-IFN-I axis pathway and protect distal organs against viral infection (45).

Interestingly, this study showed that after treatment with LPS-active MVs, the cGAS-STING-IFN-β signaling pathway in macrophages was activated. This observation was consistent with our *in vivo* data showing that the pathway was also activated in the lungs and livers of mice that received LPS-MVs. Macrophage infiltration was increased in the lungs and livers, coupled with increased levels of inflammatory cytokines and organ injury. These observations support the notion that macrophage-induced inflammatory cytokine storms are activated by mitochondrial transfer from other macrophages that are primed by inflammation.

Notably, our results showed that LPS-MVs could reprogram recipient macrophage metabolism. In particular, we observed that mitochondria derived from LPS-primed macrophages downregulated the TCA cycle and fatty acid metabolites, which are critical for sepsis. The top metabolites may be new targets for the treatment of sepsis. Our results are consistent with those of Levoux et al. to some extent, as they reported that platelets can affect the metabolic reprogramming of mesenchymal stem cells via mitochondrial transfer (32). However, our study did not identify the molecular mechanisms underlying the link between metabolic and biological functions, which is one of its limitations. Other limitations of this study include the fact that we demonstrated that metformin could reduce the overproduction of mtROS and the expressions of cGAS, STING, and IFN-β; however, we did not use metformin to treat mice injected with MVs and detect the expressions of cGAS, STING, and IFN-β. Second, although we observed metabolic reprogramming in macrophages after LPS-active MVs, we did not examine the level of differential metabolites in mice, which is the subject of ongoing work. Third, MVs have other contents aside from mitochondria, and whether these contents affect macrophages requires further investigation. Finally, many mechanisms are involved in mitochondrial transfer between cells (46–48), and the mechanisms by which mitochondria are transferred in macrophages require further investigation.

In conclusion, we demonstrated for the first time that MVs from LPS-primed macrophages were able to cause significant mitochondrial dysfunction in macrophages through mitochondrial transfer, induce macrophage M1 activation and phagocytic dysfunction, trigger cGAS-STING-IFN-β signaling activation in macrophages, and induce organ injury and systemic inflammation in mice. Metformin can inhibit the mitochondrial transfer and effects induced by LPS-MVs in macrophages. These findings suggest that mitochondrial transfer between macrophages may contribute to the inflammatory cytokine storm in sepsis.

## Data Availability

The metabolism data reported in this paper have been deposited in the OMIX, China National Center for Bioinformation/Beijing Institute of Genomics, Chinese Academy of Sciences (https://ngdc.cncb.ac.cn/omix: accession no. OMIX001234). The other data sets used and/or analyzed during the current study are available from the corresponding author on reasonable request.

## SUPPLEMENTAL MATERIAL

Fig. S1 to S5, and Tables S1 and S2 spectrum.00781-25-s0001.docx Fig. S1: BMDM could engulf microvesicles. Fig. S2: mitochondria extract from LPS-MV induced mitochondria dysfunction of receptor BMDM. Fig. S3: LPS-MV induced mitochondrial dysfunction of receptor BMDM. Fig. S4: LPS-MV could not induce M2-like macrophage phenotype. Fig. S5: LPS-MV could upregulate the expression of phosphor-STING. Table S1: primer sequences for RNA qRT-PCR. Table S2: top 25 metabolites with the highest alterations in abundance.

ASM does not own the copyrights to Supplemental Material that may be linked to, or accessed through, an article. The authors have granted ASM a non-exclusive, world-wide license to publish the Supplemental Material files. Please contact the corresponding author directly for reuse.
