# Supplementary material for: Microvesicle-transferred mitochondria trigger cGAS-STING and reprogram metabolism of macrophages in sepsis
Source: Microbiol Spectr. 2025 Sep 4;13(10):e00781-25. doi: 10.1128/spectrum.00781-25 (PMC12502667; doi:10.1128/spectrum.00781-25)
Supplement: Fig. S1 to S5, and Tables S1 and S2 — Fig. S1: BMDM could engulf microvesicles. Fig. S2: mitochondria extract from LPS-MV induced mitochondria dysfunction of receptor BMDM. Fig. S3: LPS-MV induced mitochondrial dysfunction of receptor BMDM. Fig. S4: LPS-MV could not induce M2-like macrophage phenotype. Fig. S5: LPS-MV could upregulate the expression of phosphor-STING. Table S1: primer sequences for RNA qRT-PCR. Table S2: top 25 metabolites with the highest alterations in abundance. [file spectrum.00781-25-s0001.docx]

Supplementary Materials for

**Microvesicle-transferred mitochondria trigger cGAS-STING and reprogram metabolism of macrophages in sepsis**

*
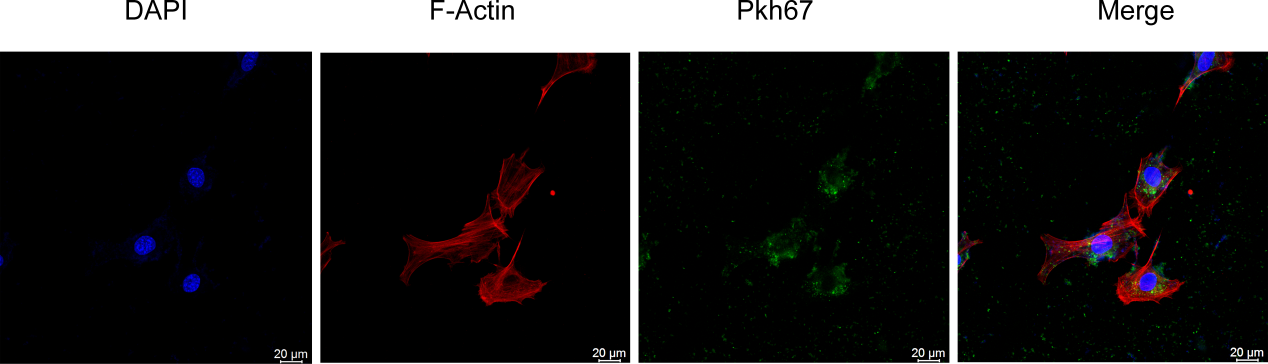
*

**Figure. S1.** BMDM could engulf Microvesicles. Blue:DAPI; Red:F-Actin; Green:Pkh67. (scale bar, 20 μm).

*
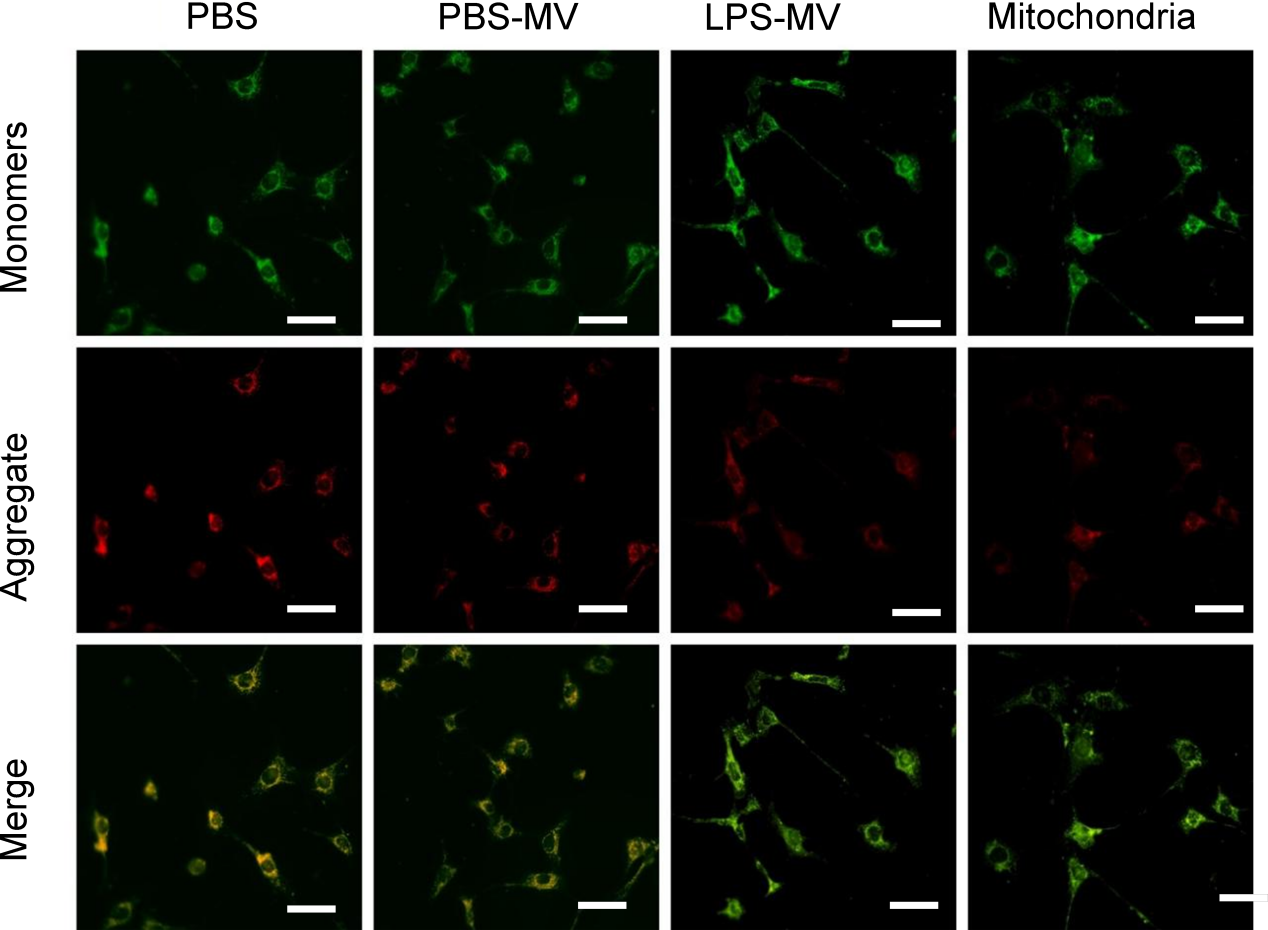
*

**Figure. S2.**Mitochondria extract from LPS-MV induced mitochondria dysfunction of receptor BMDM. Red:JC-1 Aggregate; Green:JC-1 Monomers.(scale bar, 20 μm).

*
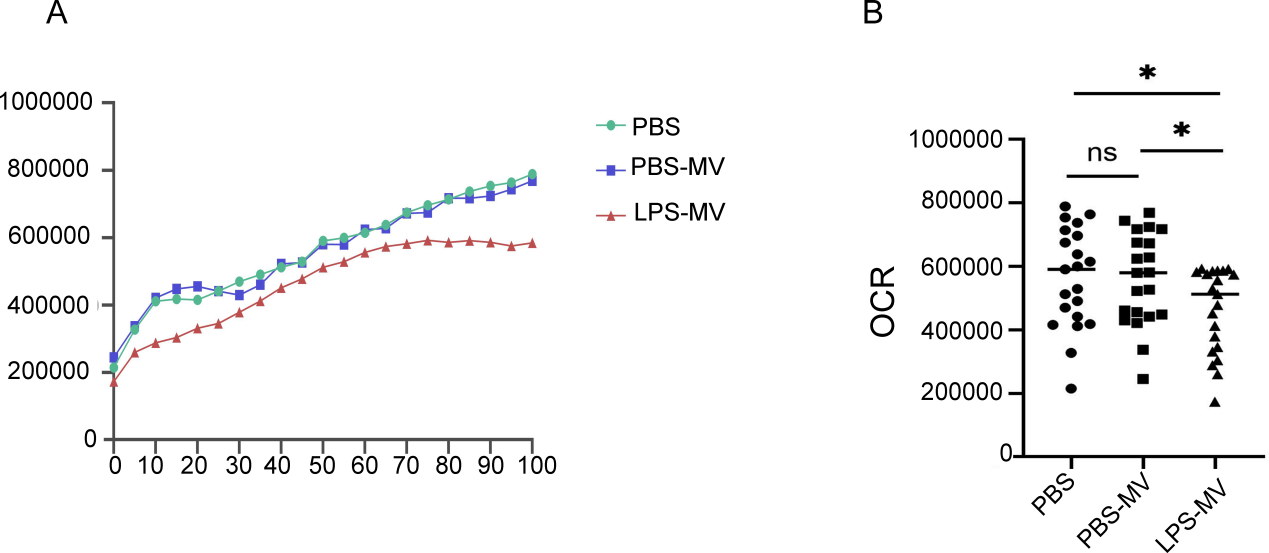
*

**Figure. S3.** LPS-MV induced mitochondria dysfunction of receptor BMDM.The mitochondrial function of BMDM was measured by OCR kit (A) and their quantitation (B). Data are means ± SEM. *P<0.05, Statistical analyses were performed using one-way ANOVA with Bonferroni's multiple comparison test.

*
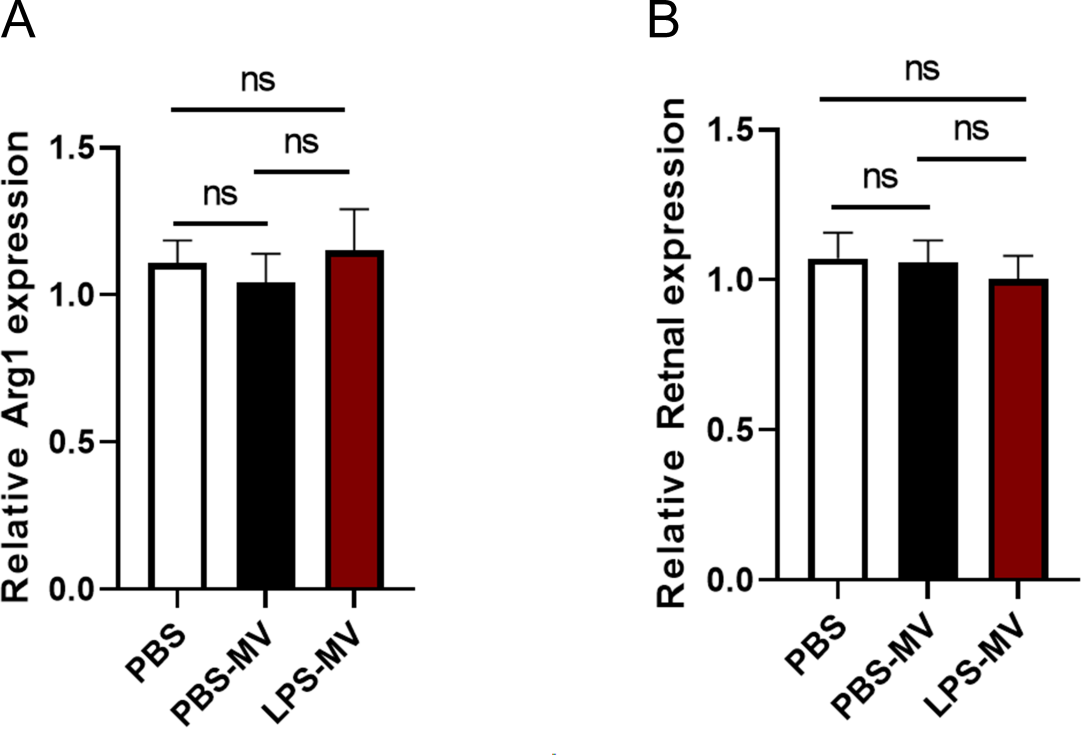
*

**Figure. S4.** LPS-MV could not induce M2-like Macrophage Phenotype. (A) The expression of Arg1 when exposed to PBS-MV, LPS-MV, or PBS was measured by qRT-PCR ; (B) The expression of Retnal when exposed to PBS-MV, LPS-MV, or PBS was measured by qRT-PCR (B). Data are means ± SEM. *P<0.05, Statistical analyses were performed using one-way ANOVA with Bonferroni's multiple comparison test.


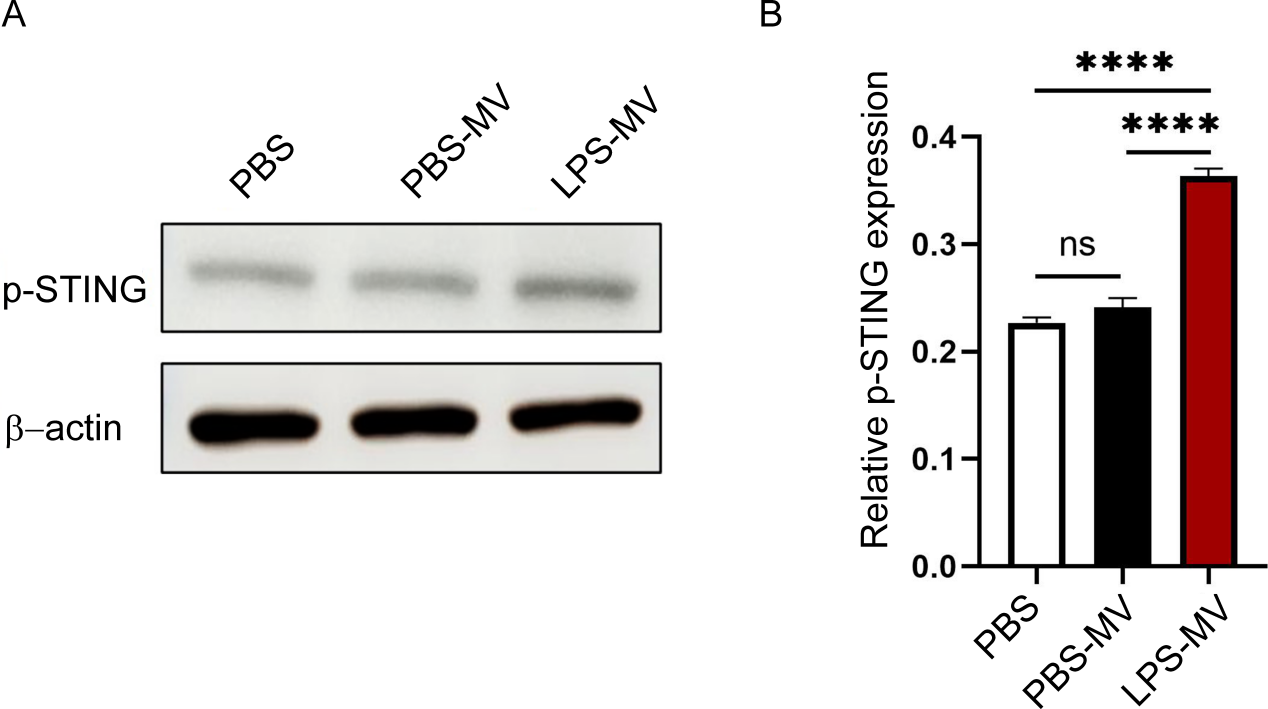


**Figure. S5.** LPS-MV could upregulate the expression of phosphor-STING. A: The p-STING expression in RAW264.7 when exposed to PBS-MV, LPS-MV, or PBS was measured by western blots (A) and their quantitation (B). Data are means ± SEM. *P<0.05, Statistical analyses were performed using one-way ANOVA with Bonferroni's multiple comparison test.


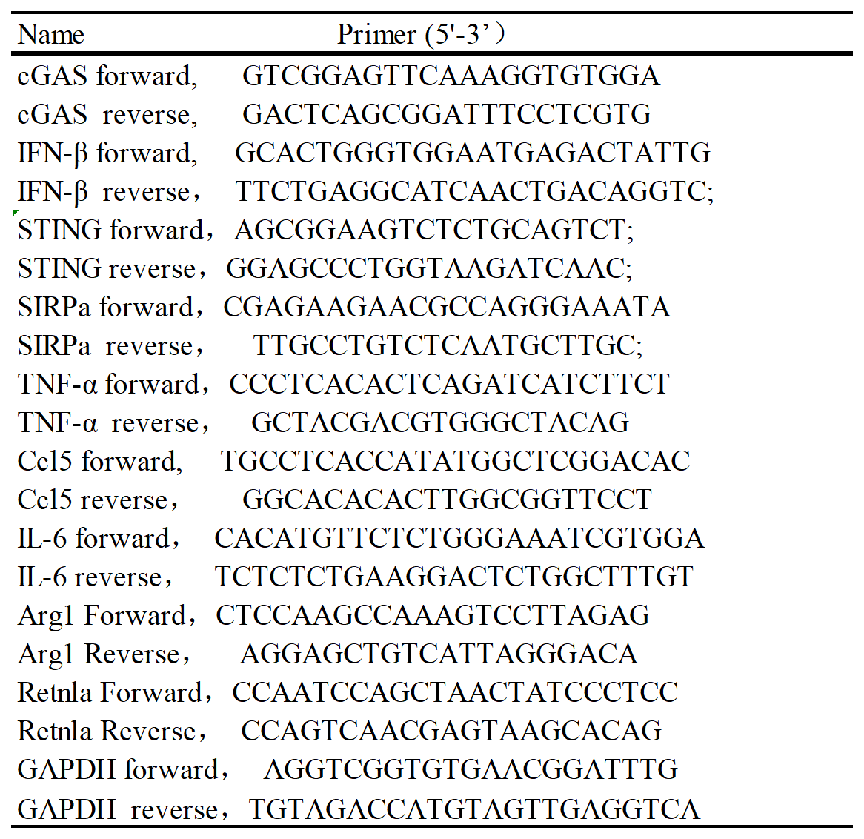


Table S1 The primer sequences for RNA qRT-PCR


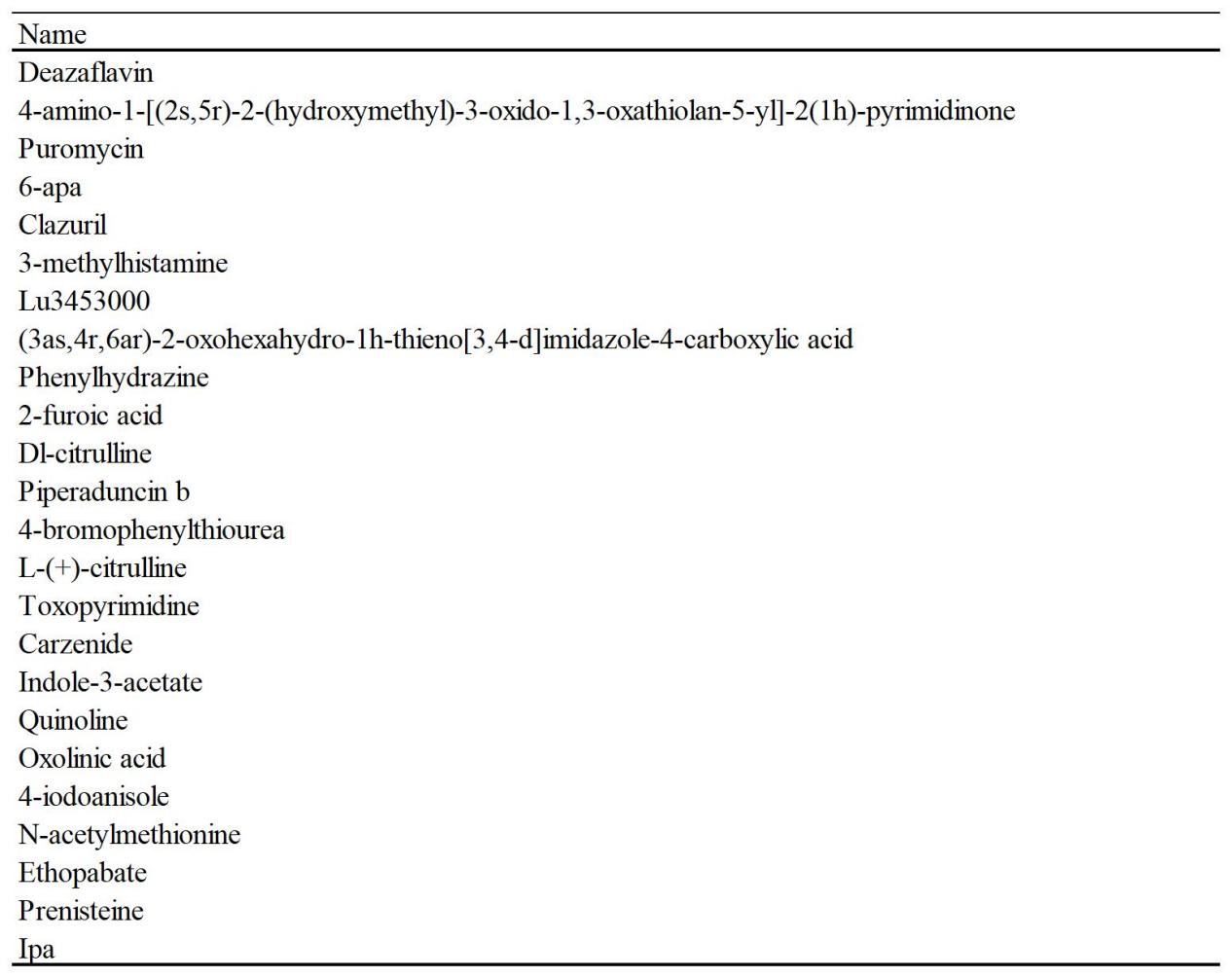


Table S2.The top 25 metabolites with the highest alterations in abundance.
